# Neck Abscess Due to Pocket Shot: Is It Just the Tip of the Iceberg?

**DOI:** 10.7759/cureus.41545

**Published:** 2023-07-07

**Authors:** Dafni Kollia, Panagiota Voukelatou, Andreas Kyvetos, Pantelitsa Elissaiou, Ioannis Vrettos

**Affiliations:** 1 2nd Department of Internal Medicine, General and Oncology Hospital of Kifissia "Agioi Anargyroi", Athens, GRC

**Keywords:** bacterial pyomyositis, mrsa, neck abscess, pocket shot, intravenous drug abusers

## Abstract

A 45-year-old Caucasian male presented to the emergency department for pain and swelling on the left side of his neck for the past 10 days. His medical history revealed that he was an intravenous (IV) drug abuser. Physical examination demonstrated a 5×5 cm red, swollen bump with a positive fluctuation on the left supraclavicular area concerning for an abscess. Fluid aspiration from the abscess was performed, and three sets of blood cultures were obtained, which later all came back positive for methicillin-resistant *Staphylococcus aureus* (MRSA). His initial blood tests revealed elevated levels of platelets, leukocytes, and C-reactive protein (CRP) and anemia. The computed tomography (CT) scan showed an enlarged pectoralis major with the presence of air. The retrosternal, infraclavicular, and supraclavicular regions also contained air. The clinical diagnosis was therefore supported by the laboratory results and imaging. Additionally, transthoracic echocardiography showed no vegetations, and transesophageal echocardiography was scheduled. Antibacterial treatment was initiated empirically from the emergency room with meropenem and vancomycin, planned for four weeks. Repeat cultures were obtained for the following three days, which were all negative. However, the patient left the hospital against medical advice and did not complete his antibiotic treatment. The risk of a peripherally inserted central catheter (PICC) line being misused for illegal narcotics was considered too high; hence, it was not recommended for continued IV antibiotic therapy at home.

Those with a history of intravenous drug use, after using the most accessible injection sites, oftentimes resort to finding alternative and potentially more dangerous injection sites. The major veins of the neck, such as the jugular, subclavian, or brachiocephalic veins, are commonly used. This technique is referred to as a "pocket shot" by intravenous drug abusers (IVDAs). Apart from the apparent abscess, clinicians should oversee for other complications including underlying pus collections, pneumothorax, mediastinitis, osteomyelitis, and hemothorax.

## Introduction

Due to intravenous (IV) drug abuse and the complications that result from it, intravenous drug abusers (IVDAs) pose a serious threat to their own lives. Apart from the direct physical harm deriving from the substance to the individual user and the tendency of the drug to induce dependence [1], IVDAs are vulnerable to both vascular injuries and a range of infections with potentially life-threatening complications, due mainly to poor hygiene and the injection of non-sterile preparations [2,3]. Among them, the most common are injection-site-related infections, such as skin and soft tissue infections, ranging from uncomplicated cellulitis and localized abscesses to life-threatening necrotizing fasciitis and severe sepsis [3].

IVDAs most frequently use accessible locations, such as veins in the upper and lower extremities, for injecting illicit drugs [4]. However, as a result of repeated injections, they experience sclerosing of the peripheral veins, and then, they frequently try to inject substances into the deep veins of the neck or the groin. The street slang for these neck injections is "pocket shots," and they are carried out in anatomical pockets between the sternal and clavicular heads of the sternocleidomastoid muscle, to gain central venous access via the subclavian and internal jugular vein or in the back of the neck's posterior triangle [5,6].

Here, we describe an IVDA young male patient presenting at the emergency department with a neck abscess due to a pocket shot.

This article was previously presented as a meeting abstract at the 20th European Congress of Internal Medicine in Malaga, Spain, on June 11, 2022.

## Case presentation

**Figure 1:**
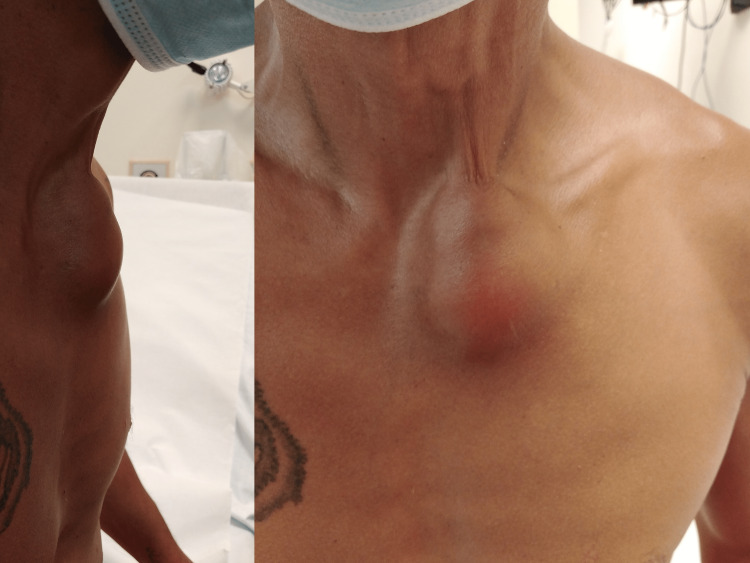
Neck abscess at the site of injection.

A 45-year-old Caucasian male presented to the emergency department complaining of pain and swelling on the left side of his neck that first started 10 days prior, along with lower limb edema. His medical history was significant for intravenous drug abuse via the internal jugular vein, mainly of heroin. His blood pressure was 103/63 mmHg with a heart rate of 91 beats/minute, and he was afebrile. Medical examination revealed a tender, warm, red, swollen area with positive fluctuation on the left supraclavicular region, with a size of about 5×5 cm (Figure 1). Other findings from the physical examination were unremarkable.

Initial laboratory tests revealed hypochromic microcytic anemia, an abnormal erythrocyte sedimentation rate, and an elevated C-reactive protein (CRP) level (Table 1).

**Table 1:** Initial laboratory tests. WBC, white blood cell; RBC, red blood cell; HCT, hematocrit; Hb, hemoglobin; MCV, mean corpuscular volume; MCH, mean corpuscular hemoglobin; PLT, platelet; ESR, erythrocyte sedimentation rate; Cr, creatinine; CRP, C-reactive protein; hs-TNI, high-sensitivity troponin I

Parameter	Value	Normal range
WBC	33.18×10^3^/μL	4.0-11.0×10^3^/μL
RBC	2.76 Μ/mL	4.5-6.3 Μ/mL
HCT	21.6%	37-48%
Hb	7 g/dL	14-18 g/dL
MCV	78.3 fL	80-96 fL
MCH	25.4 pg	27-34 pg
PLT	859 Κ/μL	150-400 Κ/μL
Ferritin	776 ng/mL	11-204 ng/mL
ESR	>130 mm/hour	2-20 mm/hour
Cr	1.1 mg/dL	0.6-1.4 mg/dL
CRP	>32 mg/dL	<0.5 mg/dL
hs-TNI	5.4 pg/mL	<11.6 pg/mL

Testing for HIV was negative, but hepatitis C virus (HCV) and anti-hepatitis B core (HBc) were positive. One unit of packed red blood cells (RBCs) was transfused raising the hemoglobin value to 9.8 g/dL.

The abscess was opened and drained, and pus cultures were obtained along with blood cultures before initiating antimicrobial treatment. He was initiated on meropenem 1 g every eight hours and vancomycin 1 g twice daily, planned for four weeks. Blood (three sets) and abscess fluid aspiration cultures were positive for methicillin-resistant *Staphylococcus aureus* (MRSA). From the antibiogram, it was sensitive to gentamicin, moxifloxacin, quinupristin/dalfopristin, linezolid, daptomycin, teicoplanin, vancomycin, tigecycline, and trimethoprim/sulfamethoxazole. Blood cultures were repeated for the following three days, after the initiation of antibiotics, which were negative.

**Figure 2:**
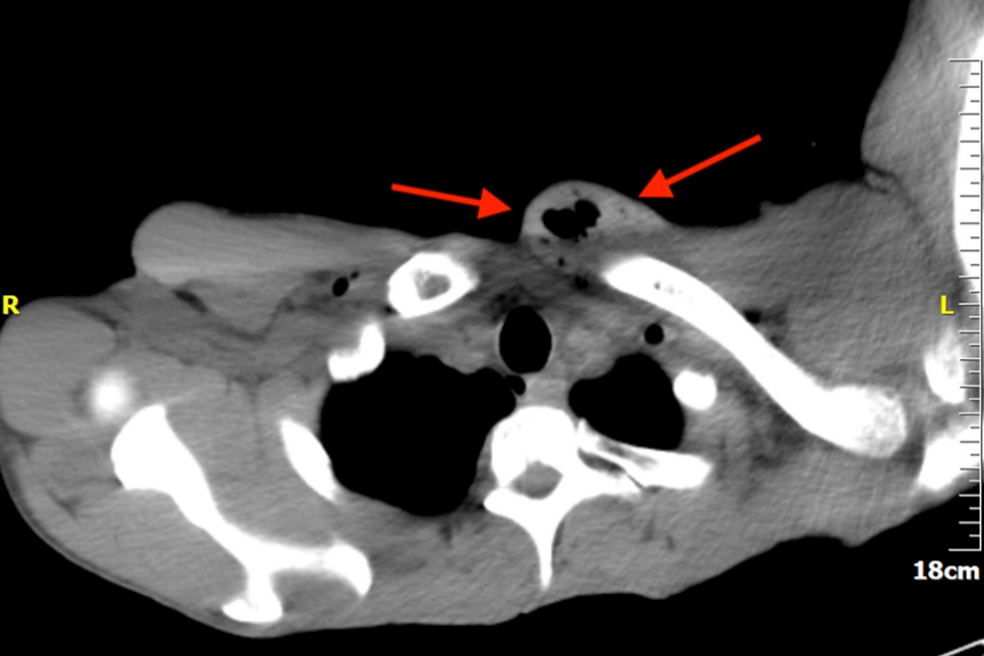
Chest CT scan, mediastinal window. CT: computed tomography

**Figure 3:**
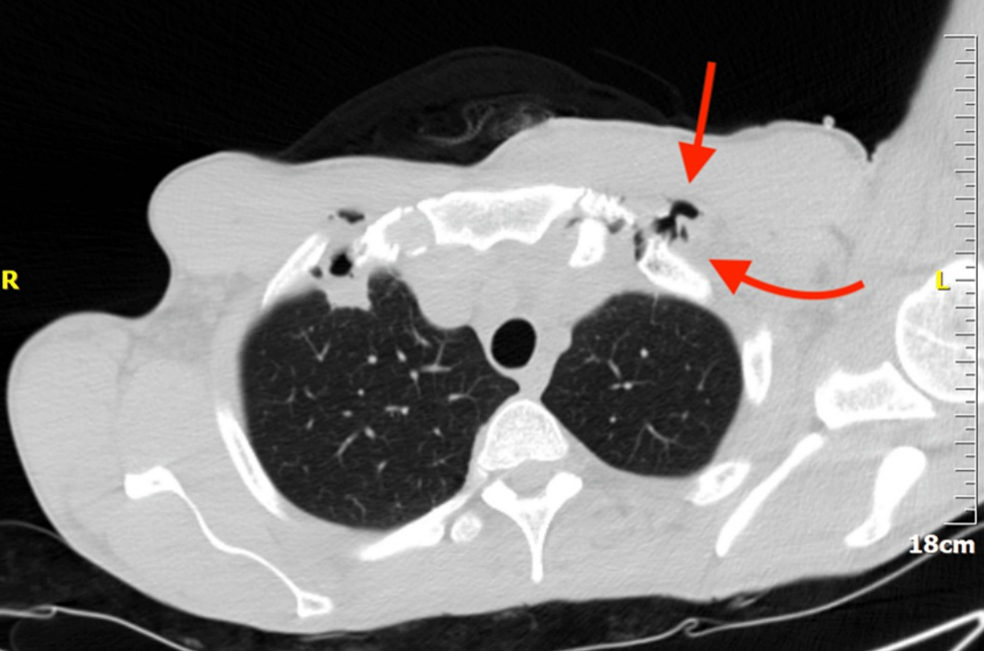
Chest CT scan, lung window. CT: computed tomography

**Figure 4:**
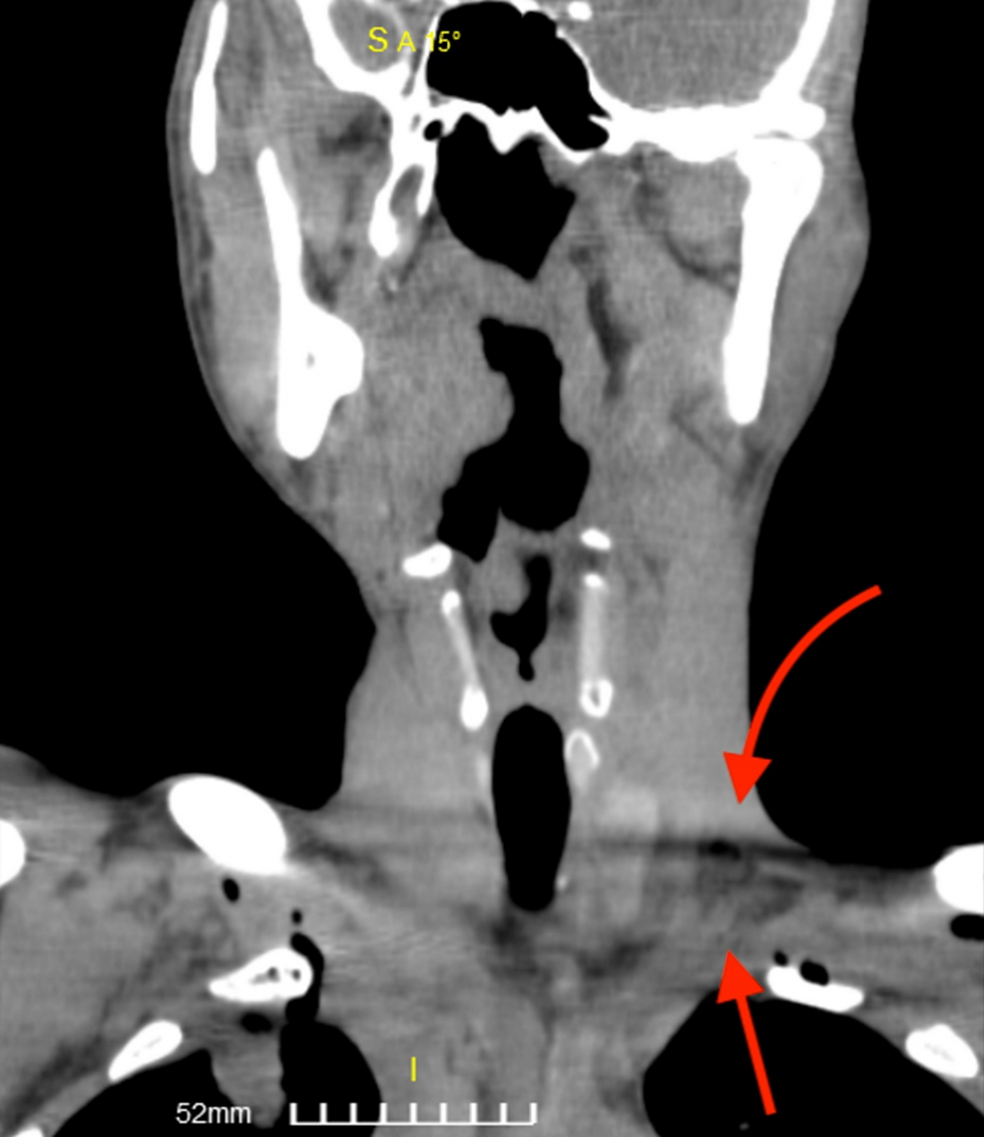
Neck CT scan, transverse view. CT: computed tomography

The computed tomography (CT) scan showed an enlarged pectoralis major with the presence of air. Air was also found in the supraclavicular, infraclavicular, and retrosternal regions (Figures 2-4). An abdominal ultrasound was performed that revealed an enlarged liver (approximate midclavicular length of 18.4 cm) and a borderline enlarged spleen with an approximate diameter of 11.2 cm. ﻿Transthoracic echocardiography was performed twice and did not reveal vegetations. It showed mildly increased left ventricular end-diastolic dimensions of 59 mm (normal range: 37-56 mm [7]) and a borderline ejection fraction of 50% (normal range: >55% [7]) without any regional wall motion abnormality and no signs of valve dysfunction. Transesophageal echocardiography was scheduled.

He was counseled multiple times by a psychiatrist for his illicit drug use and its health hazards and given instructions on how to manage withdrawal symptoms. His laboratory tests significantly improved along with the clinical improvement and the resolution of neck swelling. However, further imaging and a workup for his anemia were not conducted because the patient left the hospital against medical advice after 10 days of antibiotic therapy. A peripherally inserted central catheter (PICC) line for ongoing IV antibiotic therapy was not suggested to avoid the risk of it being used for injecting illicit drugs.

## Discussion

Our patient presented to the emergency department in an almost good clinical condition, complaining only about the swelling of his neck without reporting any other symptoms. However, he suffered from a life-threatening infection. On the contrary, in another case report of a neck abscess secondary to pocket shot intravenous drug abuse, only concomitant overlying cellulitis was referred apart from the localized abscess [4]. Nevertheless, in a literature review, central venous injections in the neck have been related to numerous localized and systemic complications. These complications could be divided into five categories: neurologic, vascular, pleuropulmonary, skeletal, and soft tissue [6]. Furthermore, IVDAs are vulnerable to a wide range of severe infections such as necrotizing fasciitis, pyomyositis, and endocarditis. Other than a thorough assessment of the injection site and the severity of localized infection, a further workup for systemic infections, especially in patients with bacteremia, is essential [3].

However, managing IVDAs can be difficult, particularly due to their frequent aggressive behavior, drug use while receiving medical care, and early self-discharge [3]. Indeed, our patient left the hospital before the diagnostic workup and the course of antibiotics were completed.

## Conclusions

This case demonstrates the utility of keeping abscesses secondary to "pocket shots" in IVDAs in the differential as missing this could lead to increased health burden and potentially life-threatening complications. A neck abscess secondary to a pocket shot in IVDAs may be the tip of the iceberg, and it must be followed by a thorough investigation for any additional localized or systemic, potentially life-threatening complications. These complications cannot be excluded by medical history and clinical examination alone. An exhaustive investigation for systemic infections is required, even in patients that showed no signs of systematic illness.
